# Are levels of DHEAS indicative of subjective health – results of the population-based longitudinal CARLA study

**DOI:** 10.1038/s41514-026-00346-0

**Published:** 2026-02-20

**Authors:** Luise Charlotte Behr, Alexander Kluttig, Andreas Simm, Rafael Mikolajczyk, Frank Bernhard Kraus, Daniel Sedding, Karin Halina Greiser, Andreas Wienke, Anne Großkopf

**Affiliations:** 1 Institute of Medical Epidemiology, Biostatistics, and Informatics, Interdisciplinary Center for Health Sciences, Medical Faculty of the Martin Luther University Halle-Wittenberg, Halle (Saale), Germany; 2 University Clinic and Outpatient Clinic for Cardiac Surgery, Medical Faculty of the Martin Luther University Halle-Wittenberg, University Medicine Halle, Halle (Saale), Germany; 3 Department of Laboratory Medicine, Unit II LM & CC - Central Laboratory, University Hospital Halle, Halle (Saale), Germany; 4 Department of Internal Medicine III, University Hospital, Martin Luther University Halle-Wittenberg, Halle (Saale), Germany; 5 Division of Cancer Epidemiology, German Cancer Research Center, Heidelberg, Germany

**Keywords:** Biomarkers, Diseases, Endocrinology, Health care, Medical research

## Abstract

Subjective and objective markers are important in describing healthy aging, yet little is known about their relationships. This study analysed the time-dependent association of dehydroepiandrosterone sulfate (DHEAS) with subjective health. At baseline, DHEAS was measured in participants aged 45–83 randomly selected from the general population. Subjective mental and physical health were assessed using the 12-item Short Form (SF-12) questionnaire at baseline and two follow-ups. In sex-specific linear regression models controlled for age, weight, tobacco consumption, Charlson Comorbidity Index, depression, and testosterone levels, the associations of DHEAS with the subscores of the SF-12 were analysed. DHEAS showed a positive cross-sectional association with subjective physical health, which was stronger in women and remained relevant after multivariable adjustment. However, longitudinal analyses revealed no long-term effect of DHEAS on subjective health. These findings suggest that the association between DHEAS and subjective physical health is temporary and that an underlying causality is unlikely.

## Introduction

The concept of healthy ageing emerged approximately sixty years ago. In the last few decades, subjective health markers, such as well-being, have become increasingly popular because they significantly impact health status^1,2^. The importance of differentiating and combining subjective and objective markers was impressively demonstrated by Strawbridge et al.: While only 18.8% of the older persons were classified as “successful agers” by a standard definition of healthy ageing based on objective markers, 50% of the participants rated themselves as ageing successfully^1^. Even if objective markers detect an “unhealthy” ageing, older people can still feel healthy. Similar discrepancies were also observed in the NRW80^+^ study, specifically regarding successful ageing in the oldest old^3^.

Most importantly, satisfaction with one’s aging is associated with decreased mortality rates independently of age, gender and illnesses^4^. A meta-analysis by Westerhof and colleagues, which included 107 studies, also found small but robust longitudinal effects of subjective aging on health and longevity^5^. Those results emphasise the importance of including subjective markers, which might more closely represent the actual reality of life for older people. However, objective measures, such as blood biomarkers and morbidity, are frequently used, partly for their ease of use. Additionally, knowledge about the causality and correlations with subjective measures is lacking. Ideally, a combination of subjective and objective markers should be chosen to obtain a comprehensive, multidimensional measure of health status or healthy aging^6^.

Dehydroepiandrosterone (DHEA), a 19-carbon steroid, is a blood biomarker of ageing. In circulation, it is present primarily in its sulfate form, dehydroepiandrosterone-sulfate (DHEAS), but the biochemical and physiological effects apply to both forms. Some studies attribute no direct hormonal function to DHEA^7^ and describe it as an inactive precursor^7–9^, which becomes active only after peripheral conversion to testosterone or oestrogen^7,10–15^. However, DHEAS can directly bind to the G-protein Gnα11^16,17^ and inhibit the Glucose-6-Phosphate-Dehydrogenase^18^, thereby affecting the pentose phosphate pathway^19^. Other effects can be explained by an antagonistic action to glucocorticoids^20–22^. DHEA secretion peaks at a younger age, with gender differences^23^. Thereafter, DHEAS levels typically decline steadily with age; however, the rate of decline varies among individuals. In some cases, levels remain stable or even increase^23–26^. Because ageing is associated with the decline of anabolic hormones^27^, DHEAS might potentially serve as a biomarker of and even have a causal role in healthy aging^7^. Roth and colleagues hypothesised that a slight decrease in DHEAS levels during ageing, as seen under caloric restriction in rhesus monkeys, would serve as a longevity marker in humans. Indeed, an age-adjusted analysis in a male-only subgroup of the Baltimore Longitudinal Aging Study (BLSA) revealed a correlation between endogenous DHEAS levels and mortality^28^. Those results could be corroborated in the Cardiovascular Health study in both sexes^7^. Furthermore, in the oldest old (All Stars), DHEAS decline was also associated with functional decline^29^. More recently, associations between DHEAS and cognitive function and depression have been reported in the English Longitudinal Aging Study (ELSA)^30,31^, as well as correlations of DHEAS with epigenetic age (Horvath’s clock) and intrinsic capacity (IC)^32,33^.

In terms of subjective health, Morales et al. first reported improved well-being under DHEAS supplementation in both sexes^34^. While slight improvements in well-being were observed in women with adrenal insufficiency and patients with Addison’s disease^35,36^, other authors could not find an improvement in subjective health or well-being in healthy individuals undergoing DHEAS supplementation^36–41^.

While previous studies suggest there might be a connection, the current data on the association between DHEAS and subjective health is limited and inconclusive. Additionally, in the studies of Roth et al., Van Niekerk et al., Flynn et al. and Arlt et al.^28,38–40^, all participants were men, leading to a less detailed understanding of the association between DHEAS and subjective health in women. Furthermore, previous research primarily focused on DHEAS supplementation from 50 to 100 mg/d. These studies do not allow any conclusions about the effects of physiological DHEAS levels from an endogenous production of 10–25 mg/d^24^. Our study offers new insights, especially in these two areas, as it includes both sexes, observes the effects of physiological DHEAS levels, and analyses the long-term effect of the association. The long follow-up period of our population-based cohort study allows us to evaluate the association between baseline DHEAS and subjective health both cross-sectionally and over a 9-year follow-up period. We test the hypothesis that higher DHEAS levels are associated with better subjective health both cross-sectionally and longitudinally.

## Results

### Study population

**Fig. 1:**
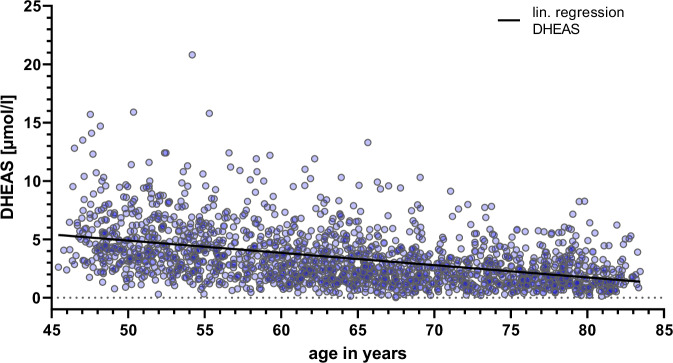
DHEAS levels decrease with increasing age in the CARLA study population. Alt text: Graph depicting DHEAS values of the study participants, showing a linear decline with age.

The baseline characteristics of the study population show that men had higher DHEAS and testosterone levels, smoked more, and were more often full-time employees (Table 1). Women were more likely to be physically active, but also more likely to live with depressive symptoms than men were. Other categories showed only marginal differences between the sexes. Furthermore, our study population showed lower DHEAS levels among older participants, both females and males (Fig. 1).

**Table 1:** Baseline characteristics of the study population

	Men (*N* = 967, 54.4%)			Women (*N* = 812, 45.6%)		
	Mean	SD	Range (min; max)	Mean	SD	Range (min; max)
Age in years	65	±10	45; 83	64	±10	45; 83
Weight in kg	84	±14	44; 145	73	±15	41; 151
BMI in kg/m²	28	±4	17; 43	29	±5	18; 54
Number of smoked cigarettes, cigars, pipes/day	3.8	±8.8	0; 60	1.9	±5.4	0; 40
Employment status *N* (%)						
Full time	245	(25.3)		150	(18.5)	
Regular part-time	19	(2)		50	(6.2)	
Marginal/irregularly employed	36	(3.7)		31	(3.8)	
None	667	(69)		580	(71.5)	
Sport, yes *N* (%)	286	(30.6)		347	(42.8)	
Charlson Comorbidity Index	1.69	±2.25	0; 17	1.74	±2.18	0; 13
Depression score above 23, yes *N* (%)	42	(4.4)		85	(10.7)	
DHEAS in µmol/L	4.04	±2.67	0.28; 20.80	2.59	±1.76	0.02; 12.40
Testosterone in nmol/L	14.84	±5.94	0.19; 50.40	0.57	±0.51	0.09; 8.88
Subjective mental health	53.42	±9.2	6.08; 71.70	49.82	±11.4	7.27; 70.29
Subjective physical health	45.57	±9.9	14.92; 63.94	43.73	±10.7	15.71; 67.59

*SD* standard deviation, *min* minimum, *max* maximum.

### Time-dependent association Of DHEAS with subjective health

DHEAS levels at baseline showed no association with the mental component scale (MC) of the SF-12 at baseline in unadjusted (Model I) as well as adjusted linear regression models (Model II and III). We also found no longitudinal association between DHEAS levels at baseline and subjective mental health measured at follow-up 1 and 2.

**Fig. 2:**
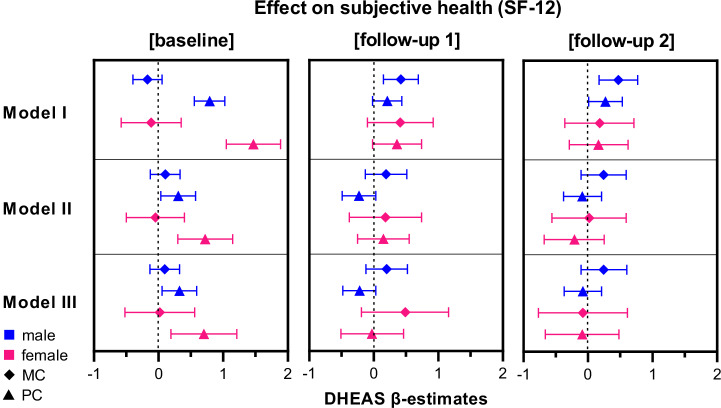
β-Estimates (symbols) and 95% confidence intervals (CI: bars) of baseline DHEAS for the subjective health in a time-dependent analysis of male (blue) and female (magenta) participants. MC: mental component scale of the SF-12 (diamond), PC: physical component scale of the SF-12 (triangle), Model I is unadjusted; Model II is adjusted for age, weight, tobacco consumption, CCI and depressive symptoms, and Model III contains an additional adjustment for testosterone. Alt Text: Graphical representation of the effects of baseline DHEAS on subjective health in the baseline and follow-ups.

In contrast, the employed models revealed a positive association of DHEAS with the physical component (PC) of the SF-12 at baseline in both male and female participants in all three models (Fig. 2). Effect-sizes were larger in females than in males (Model I: *β*_male_ = 0.79 (95% CI 0.5–1. 03), *β*_female_ = 1.47 (95% CI 1.05–1.89); Model II: *β*_male_ = 0.30 (95% CI 0.04–0.57), *β*_female_ = 0.72 (95% CI 0.3–1.15)) and changed only slightly with the inclusion of testosterone (Model III: *β*_male_ = 0.32 (95% CI 0.05–0.59), *β*_female_ = 0.70 (95% CI 0.19–1.21)).

No effects for baseline DHEAS were found in longitudinal models with PC in follow-ups 1 and 2 as outcomes (Table 2, Fig. 2).

**Table 2:** Estimates of effects of baseline DHEAS and baseline covariates on subjective health, at baseline, follow-up 1, and follow-up 2

Baseline	Mental component		Physical component		
Model III	Men (*N* = 887)	Women (*N* = 606)	Men (*N* = 887)		Women (*N* = 606)
DHEAS (95%CI)	0.1 (−0.13; 0.32)	0.02 (−0.52; 0.56)	0.32 (0.05; 0.59)		0.70 (0.19; 1.21)
Testosterone	0.04 (−0.05; 0.13)	0.42 (−1.24; 2.07)	0.12 (0.01; 0.22)		−0.18 (−1.75; 1.38)
Age	0.14 (0.07; 0.21)	0.04 (−0.05; 0.14)	−0.10 (−0.18; −0.03)		−0.20 (−0.29; −0.11)
Weight	0.04 (−0.00; 0.07)	0.03 (−0.02; 0.09)	−0.01 (−0.18; 0.03)		−0.11 (−0.16; −0.06)
Tobacco consumption	0.04 (−0.02; 0.11)	0.00 (−0.15; 0.15)	−0.06 (−0.13; 0.01)		−0.08 (−0.22; 0.06)
Charlson Comorbidity Index	−0.18 (−0.45; 0.07)	0.18 (−0.23; 0.59)	−1.37 (−1.68; −1.07)		−1.18 (−1.57; −0.79)
Depressive symptoms	23.37 (20.85; 25.89)	19.35 (16.73; 21.96)	1.32 (−1.63; 4.27)		3.5 (1.02; 5.97)

Follow-up 1	Mental component		Physical component	
Model III	Men (*N* = 699)	Women (*N* = 439)	Men (*N* = 699)	Women (*N* = 439)
DHEAS	0.2 (−0.12; 0.52)	0.49 (−0.19; 1.16)	−0.22 (−0.48; 0.03)	−0.03 (−0.51; 0.46)
Subjective health baseline score	0.44 (0.35, 0.54)	0.41 (0.31; 0.52)	0.53 (0.46; 0.59)	0.50 (0.42; 0.58)
Testosterone	0.00 (−0.13; 0.13)	−0.44 (−2.39; 1.52)	0.08 (−0.03; 0.18)	0.32 (−1.07; 1.7)
Age	−0.09 (−0.19; 0.00)	0.00 (−0.13; 0.13)	−0.21 (−0.28; −0.13)	−0.16 (−0.25; −0.07)
Weight	0.01 (−0.05; 0.06)	-0.02 (-0.09; 0.05)	−0.04 (−0.08; 0.01)	−0.06 (−0.11; −0.01)
Tobacco consumption	−0.08 (−0.17; 0.01)	−0.02 (−0.21; 0.16)	−0.09 (−0.16; −0.02)	−0.03 (−0.16; 0.10)
Charlson Comorbidity Index	−0.71 (−1.13; −0.28)	−0.52 (−1.06; 0.02)	−0.81 (−1.16; −0.46)	−0.38 (−0.77; 0.01)
Depressive symptoms	2.70 (−1.37; 6.77)	5.58 (1.41; 9.76)	1.31 (−1.42; 4.05)	4.63 (2.00; 7.26)

Follow-up 2	Mental component		Physical component	
Model III	Men (*N* = 553)	Women (*N* = 375)	Men (*N* = 553)	Women (*N* = 375)
DHEAS	0.24 (−0.11; 0.60)	−0.08 (−0.77; 0.61)	−0.08 (−0.37; 0.21)	−0.09 (−0.66; 0.48)
Subjective health baseline score	0.43 (0.32; 0.54)	0.30 (0.19; 0.40)	0.45 (0.37; 0.53)	0.40 (0.31; 0.49)
Testosterone	0.07 (−0.08; 0.22)	0.58 (−1.32; 2.48)	0.10 (−0.03; 0.22)	−0.05 (−1.60; 1.50)
Age	−0.09 (−0.20; 0.02)	−0.05 (−0.19; 0.09)	−0.17 (−0.26; −0.08)	−0.33 (−0.45; −0.22)
Weight	−0.02 (−0.08; 0.04)	−0.01 (−0.08; 0.07)	−0.03 (−0.08; 0.02)	−0.09 (−0.16; −0.03)
Tobacco consumption	−0.07 (−0.17; 0.02)	0.05 (−0.14; 0.23)	−0.06 (−0.14; 0.02)	−0.10 (−0.25; 0.05)
Charlson Comorbidity Index	−0.77 (−1.30; −0.26)	−0.53 (−1.11; 0.05)	−0.72 (−1.16; −0.27)	−0.24 (−0.73; 0.24)
Depressive symptoms	1.87 (−3.14; 6.88)	8.07 (3.80; 12.35)	0.68 (−2.85; 4.21)	5.68 (2.58; 8.78)

95% confidence interval (95% CI) in brackets.

Of the covariates considered, the baseline score of subjective health, comorbidities at the baseline examination in men, and depressive symptoms in women show a longitudinal effect on subjective health (Table 2). Unsurprisingly, age and, to a lesser extent, weight also have a longitudinal effect on subjective physical health.

## Discussion

Our research showed that DHEAS at baseline was associated with the physical component of subjective health only in cross-sectional but not in longitudinal analyses. This effect was more pronounced in women. An effect of DHEAS on the mental component of the SF-12 could not be found in either men or women.

Most previous studies concerning DHEAS and its effects on subjective health or well-being analysed the impact of short- to medium-term supplementation. While slight improvements in well-being were observed in women with adrenal insufficiency^35^ and patients with Addison’s disease under DHEAS-substitution^36^, most studies on healthy participants did not report significant impacts^38–40^. Baulieu et al. reported improved skin, possibly leading to a changed self-perception in the subjects^42^. The DAWN trial showed increased satisfaction with life-scores but no changes in SF-36^41^. In contrast, this study evaluates the effects of physiological levels of DHEAS instead of pharmacologically elevated levels. Previously, differences in the effects of DHEAS in pharmacological compared to physiological doses were discussed^43^.

An association of DHEAS with physical subjective health could only be found in our cross-sectional analysis, and it was more pronounced in women. This suggests that if there is a causal effect, it only exists in the short term. In other studies, observing associations of physiological DHEAS levels and aspects of (healthy) ageing, effects are also often cross-sectional and small. Higher DHEAS levels were reported to be associated with better cognition^30^ and lower biological age in men^32^ and depressive symptoms in both sexes^31^. In addition, lower DHEAS correlated with lower intrinsic capacity (IC)^33^. Taken together, those previous results point in the same direction as the results of this study, indicating overall better outcomes with higher DHEAS levels. The IC, which includes measures of cognition, locomotion, vitality, psychology, sensory function, as well as biological age, could even be a mediator of the results of the SF-12 reported in this study. Furthermore, it might be interesting to consider depressive symptoms not as a confounder, but a mediator of DHEAS effects on the MC of the SF-12, given the direct association shown by others and the strong effect seen in our study.

The lack of longitudinal associations in our study argues against a causal effect of DHEAS on subjective health. Instead, if DHEAS does indeed have a relevant role in healthy ageing, it may represent an aspect of healthy ageing that is independent of subjective health.

Although no effects were found in the analyses of the follow-ups, the longitudinal dataset is an advantage of our study, as it allows for more causal conclusions. Previous studies have demonstrated that DHEA(S) levels and their progression show broad inter- and intra-individual variability at different ages^9^ and that DHEAS-trajectories might be most indicative^7,29^. The analysis of courses of DHEAS levels and associations with healthy ageing is an auspicious task for further research with our data on these grounds. On the other hand, the time between measuring the exposure and the outcome of an average of four years (follow-up 1) and nine years (follow-up 2) leaves much leeway for unknown influencing factors.

Additionally, the broad age spectrum of the participants is an advantage of the present study. Most previous studies included participants starting at the age of 65, while in this work, the youngest participants were 20 years younger than this classical cut-off. Therefore, important information about aging processes in younger people and comparisons between different age groups can be gained, especially because interventions on aging, like lifestyle adjustment, showed the best results when applied as early as possible.

When describing the effects of DHEAS on subjective health, the relevance of the identified effects must also be discussed critically. In an intervention study of total knee arthroplasty, the minimally clinically important difference (MICD), perceived as beneficial or harmful, was 1.8 for the PC and 1.5 for the MC of SF-12^44^. Applied to our study, such a difference in subjective health would need a difference of DHEAS levels of at least 4.7 µmol/l (regarding our most substantial effect of women in physical health in Model III), corresponding to about two and a half standard deviations. Other studies report higher or lower MCID, depending on the exposure variable^45,46^. However, results from intervention studies that supplement DHEA might not apply directly to our observational data, as they usually require larger or more consistent outcome differences than longitudinal studies of the same individuals.

This study has some further limitations that warrant consideration. Due to loss-to-follow-up, the participant numbers steadily decline, lowering the statistical power. Furthermore, DHEAS levels were only determined at baseline, thus lacking information on the trajectory of change over time. As trajectories are potentially more indicative of the biological ageing process, the interpretability of DHEAS in this regard is limited.

Even after adjusting for testosterone, it is unclear whether the observed effects might be due to metabolites of DHEAS or other hormones. Oestrogen was not determined in this study, limiting the results’ explanatory power. Since previous studies showed sex-specific effects of DHEAS and suggested an action depending on the hormonal milieu of the individual^29,37,47,48^, analyses of the participants’ hormonal conditions could have helped explain the observed effect we found predominantly for women.

Additionally, the study population showed a high proportion of persons at risk for diseases, which may explain low subjective health ratings and limit the transferability to younger, healthier populations. Because comorbidity can affect DHEAS levels and subjective health, we adjusted our results for comorbidity via the CCI. In an additional analysis, we constructed a healthier study population by including only participants who did not live with cancer, chronic heart failure, coronary heart disease, cardiovascular disease, or diabetes at baseline. This analysis revealed no significant modification of the results compared to the previously presented analysis (data not shown).

Overall, our results show that DHEAS is only related to physical subjective health when measured simultaneously, with the effect being more substantial in women. However, DHEAS cannot be used to predict future subjective health and changes therein. Nevertheless, in addition to subjective health, DHEAS could also be an indicator of other aspects of healthy ageing.

Thus, even though DHEAS was previously described as an objective marker of healthy ageing, it does not correlate strongly with subjective health or possess predictive potential in this study. A causal link between DHEAS and subjective health could not be seen in our study, as indicated by the absence of longitudinal effects. Thus, the research hypothesis, which presumes an association in both the short and long term, needs to be partly rejected. Nevertheless, with our study’s prospective and observational perspective, we gained fundamental insights into the association between physiological DHEAS levels and subjective health, which can inform future experimental studies. As no longitudinal association between DHEAS and subjective health was observed, these findings suggest that DHEAS may represent one facet of a favourable ageing profile rather than exerting a direct effect on subjective health status. Finally, combining measurements of DHEAS or other biomarkers with subjective health assessments might help cover multiple aspects of the multidimensional concept of healthy ageing.

## Methods

### Study design

Data from the baseline examination and the two follow-ups of the CARLA study, a population-based epidemiological cohort study^49^ in Halle (Saale), Germany, were used for this analysis. The study population consisted of a random representative sample of 3437 invited men and women between 45 and 80 years old at baseline, of which 1779 participants took part in the baseline exams. The first follow-up occurred after a mean of 4 years with 1436 participants, and the second follow-up after 8.8 years with 1136 participants^50^. Despite the high baseline response, the participants in the CARLA study differed from the general population in terms of their higher socioeconomic status^50^. A more detailed description of CARLA can be found elsewhere^50,51^. Ethics approval was granted by the Ethics Committee of the Medical Faculty of the Martin Luther University Halle-Wittenberg for the baseline and follow-up examinations (register number 164/12.10.05/1). All participants gave written informed consent. All research involving human research participants, material, and data was performed in accordance with the Declaration of Helsinki.

### Outcome subjective health

To assess subjective health, we used the SF-12 Health Survey^52^, a simplified version of the SF-36, a valid instrument for use in surveys of older people^53^. The SF-36 and SF-12 cover eight health domains: physical functioning, social functioning, limitations of role activities because of physical health, limitations of role activities because of emotional problems, bodily pain, general health, mental health and vitality^54^. In our survey, SF-12 questionnaires were sent to the participants. Information from all 12 items was weighted from the German total population of 1994 and used to calculate the SF-12 physical component (PC) and mental component (MC) scales. The data were collected at baseline and the first two follow-ups of this study.

### Exposure DHEAs

Since DHEA concentrations can fluctuate considerably over the day, we used DHEAS as a biomarker instead, given its higher diurnal concentration stability^55^. DHEAS was measured in venous blood samples (non-fasting) at baseline, which were centrifuged after venipuncture and stored at −80 °C until measurement. In 2020, DHEAS was measured in these stored baseline samples. Determination was carried out at the Central Laboratory of the University Hospital Halle (Saale) with a Roche Elecsys® DHEA-S ECLIA (electrochemiluminescence immunoassay) on a Roche cobas e801 analytical platform. The unit of measurement was µmol/L.

### Assessment of covariables

As described in previous studies, levels of DHEAS can be influenced by various factors and tend to show oestrogenic or androgenic effects depending on hormonal and metabolic conditions^43^. At the same time, these factors can be associated with subjective health. Therefore, we included the following covariables in our analyses in addition to age and sex.

Weight in kilograms was assessed by weighing every participant at the baseline examination. Tobacco consumption was measured as the number of cigarettes, cigars, or pipes currently smoked per day, determined via self-disclosure of the participants at baseline.

The comorbidity of the study population was assessed by a modified Charlson Comorbidity Index (CCI)^56,57^, which considers the number and severity of diseases and was modified to include additional medical conditions in our data. Our version of the comorbidity index consists of the following items and their dedicated weighting: asthma or chronic bronchitis (1), osteoarthritis or rheumatoid arthritis (1), stroke (1), peripheral arty disease (1), stomach diseases (1), diabetes mellitus (2), coronary heart disease and heart failure (2), kidney diseases (2), liver diseases (3), cancer (6). The score encompasses a range from 0 to 20. The assessment of comorbidities was based on information from current medication, electrocardiogram, echocardiography, and results of markers in blood samples or via standardised interviews and self-assessments.

Depressive symptoms were assessed at baseline using the CES-depression-score with a range of 0 to 60. We dichotomised the score at a cut-off of ≥23 to distinguish persons with depressive symptoms from those not fulfilling this criterion.

As described above for DHEAS, testosterone was measured in frozen, non-fasting venous blood samples at baseline in the Central Laboratory of the University Hospital Halle (Saale) with a Roche Elecsys® Testosterone ECLIA (electrochemiluminescence-immunoassay) on a Roche cobas e801 analytical platform. The unit was nmol/L.

### Statistical analysis

We calculated three sex-specific models per analysis, each for cross-sectional (baseline), 4- and 9-year follow-up data of the SF-12, to analyse both the potential cross-sectional and long-term effect of baseline DHEAS on subjective mental and physical health. Participants with missing data points were excluded from the analysis, and numbers are reported in Table 2. In the first model, the univariable association of DHEAS with subjective health was analysed separately for both subjective mental and subjective physical health. In a second model, the association between DHEAS and subjective health was adjusted for baseline covariables: age, weight, tobacco consumption, CCI and depressive symptoms. Again, analysis was conducted separately for mental and physical health. A third model was used to control for a possible mediating effect of testosterone by including baseline testosterone as an additional covariable to the ones of Model II.

With our sample size, we can estimate the effect of DHEAS on subjective health with a two-sided 95% confidence interval width of one unit on the subjective health scale. Since previous studies declared 1.5 for physical and 1.8 for mental units of the subjective health scale as a minimally relevant effect^44^, the number of cases can be assumed to be sufficient.

All models were sex-stratified. Statistical analyses were performed using SAS version 9.4 (SAS Inc., Cary, North Carolina, USA).

## Data Availability

Researchers interested in a potential collaboration can apply for the data by sending an email to [carlastudie@uk-halle.de] or by submitting a form that is available on the CARLA study website https://webszh.uk-halle.de/carla-studie/. A formal application must be submitted to access the data with a detailed research proposal consisting of a title, authors, research questions, a brief scientific background, a list of needed variables, and proposed statistical analyses. The CARLA Study steering committee will review all proposals, and a final decision on the use of data will be made.
